# Orientational Disorder and Molecular Correlations in Hybrid Organic–Inorganic Perovskites: From Fundamental Insights to Technological Applications

**DOI:** 10.1021/acsami.4c12762

**Published:** 2024-12-24

**Authors:** Carlos Escorihuela-Sayalero, Ares Sanuy, Luis Carlos Pardo, Claudio Cazorla

**Affiliations:** † Group of Characterization of Materials, Departament de Física, Universitat Politècnica de Catalunya, Campus Diagonal-Besòs, Av. Eduard Maristany 10−14, Barcelona 08019, Spain; ‡ Research Center in Multiscale Science and Engineering, Universitat Politècnica de Catalunya, Campus Diagonal-Besòs, Av. Eduard Maristany 10−14, Barcelona 08019, Spain

**Keywords:** hybrid organic–inorganic perovskites, molecular dynamics simulations, molecular rotational dynamics, molecular correlations, entropy calculations

## Abstract

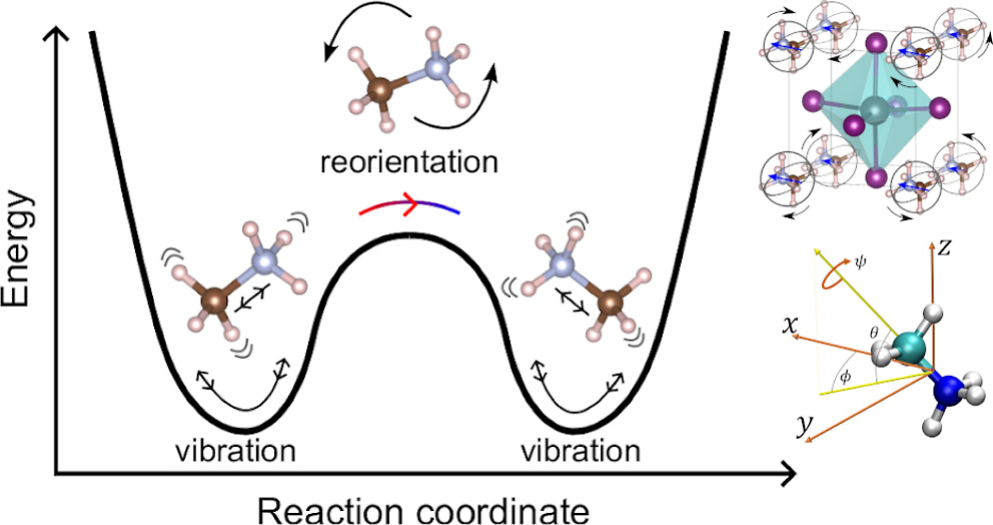

Hybrid organic–inorganic perovskites (HOIP) have emerged in recent years as highly promising semiconducting materials for a wide range of optoelectronic and energy applications. Nevertheless, the rotational dynamics of the organic components and many-molecule interdependencies, which may strongly impact the functional properties of HOIP, are not yet fully understood. In this study, we quantitatively analyze the orientational disorder and molecular correlations in archetypal perovskite CH_3_NH_3_PbI_3_ (MAPI) by performing comprehensive molecular dynamics simulations and entropy calculations. We found that, in addition to the usual vibrational and orientational contributions, rigid molecular rotations around the C–N axis and correlations between neighboring molecules noticeably contribute to the entropy increment associated with the temperature-induced order–disorder phase transition, Δ*S*_*t*_. Molecular conformational changes are equally infrequent in the low-*T* ordered and high-*T* disordered phases and have a null effect on Δ*S*_*t*_. Conversely, the couplings between the angular and vibrational degrees of freedom are substantially reinforced in the high-*T* disordered phase and significantly counteract the phase-transition entropy increase resulting from other factors. Furthermore, the tendency for neighboring molecules to be orientationally ordered is markedly local, consequently inhibiting the formation of extensive polar nanodomains at both low and high temperatures. This theoretical investigation not only advances the fundamental knowledge of HOIP but also establishes physically insightful connections with contemporary technological applications like photovoltaics, solid-state cooling, and energy storage.

## 1 Introduction

Hybrid organic–inorganic perovskites (HOIP) are solids with the chemical formula ABX_3_, where A and B–X represent organic and inorganic ions, respectively. Analogous to oxide perovskites, HOIP exhibits high-temperature crystalline phases with cubic symmetry. HOIP have emerged as a promising family of optoelectronic and energy materials, with significant potential for use in photovoltaic and light-emitting devices,^1−4^ field-effect transistors,^5−7^ energy storage,^8−10^ and solid-state refrigerators,^11−14^ among other technologies. Methylammonium lead iodide, CH_3_NH_3_PbI_3_ (MAPI), is an archetypal HOIP that has been extensively investigated for solar cells and quantum dots applications.^15−19^

A characteristic physical trait of HOIP is that, upon increasing temperature, they undergo order–disorder phase transitions involving orientational molecular disorder, which on average gives rise to the high-symmetry cubic lattice.^20^ Interestingly, the orientational dynamics of the organic cations may profoundly affect the functional properties and lattice dynamics of HOIP.^21−25^ To cite few examples, the contribution of the molecular CH_3_NH_3_^+^ (MA^+^) rotations to the static dielectric response of MAPI has been estimated to be as large as ∼40%.^26^ The origin of the hysteresis frequently observed in photocurrent–voltage measurements of MAPI-based solar devices, which offers promise for memristors and nonvolatile memory applications,^27^ is also thought to be related to the rotational dynamics of molecular cations.^28^ Additionally, the contribution of the orientational MA^+^ degrees of freedom to the caloric response of MAPI, as driven by external bias like hydrostatic pressure and electric field shifts, has been shown to be substantial.^29,30^

Despite these advancements, there remains a fundamental lack of understanding regarding the correlations between organic–organic and organic–inorganic ions, and how these correlations may impact the molecular orientational dynamics and, in turn, the functional properties of HOIP. For instance, it has been experimentally shown for MAPI that, even at temperatures well below the order–disorder phase transition point, the organic cations remain highly disordered and mobile.^31,32^ Conversely, MA^+^ reorientational motion is largely inhibited in mixed-halide hybrid perovskites such as MAPbIBr_2_ and MAPbI_2_Br.^33^ The role of molecular correlations in the potential formation of ordered molecular domains also remains debatable.^26,28,34^ Similarly, previous studies assessing the existence of caloric effects in HOIP have mostly considered organic cations as independent entities, thus neglecting the likely influence of many-molecule interdependencies on the observed solid-state cooling figures of merit.^11−14,30^

In this study, we delve into the analysis of molecular disorder and likely organic–organic and organic–inorganic ionic correlations in HOIP by conducting extensive molecular dynamics (MD) simulations for MAPI. Our quantitative investigations focus on evaluating the entropy change associated with the temperature-induced order–disorder phase transition occurring near room temperature (i.e., between the low-*T* tetragonal *I*4/*mcm* and high-*T* cubic *Pm*3̅*m* phases^35,36^), Δ*S*_*t*_, which is a physically interpretable and readily measurable quantity. We also address through MD simulations the question about the possible formation of ordered MA cation nanodomains at finite temperatures. Additionally, we establish physically insightful connections between our fundamental findings and technological applications of current interest like photovoltaics, solid-state cooling, and energy storage.

The organization of this article is as follows. First, we present a detailed description of the simulation approach employed for the evaluation of Δ*S*_*t*_. Next, we report our computational results along with some discussions and subsequently provide a summary of the main conclusions. The technical details of our classical MD simulations and entropy calculation approach can be found in the Methods section.

## 2 Computational Approach

### 2.1 MD Simulations

**Figure 1:**
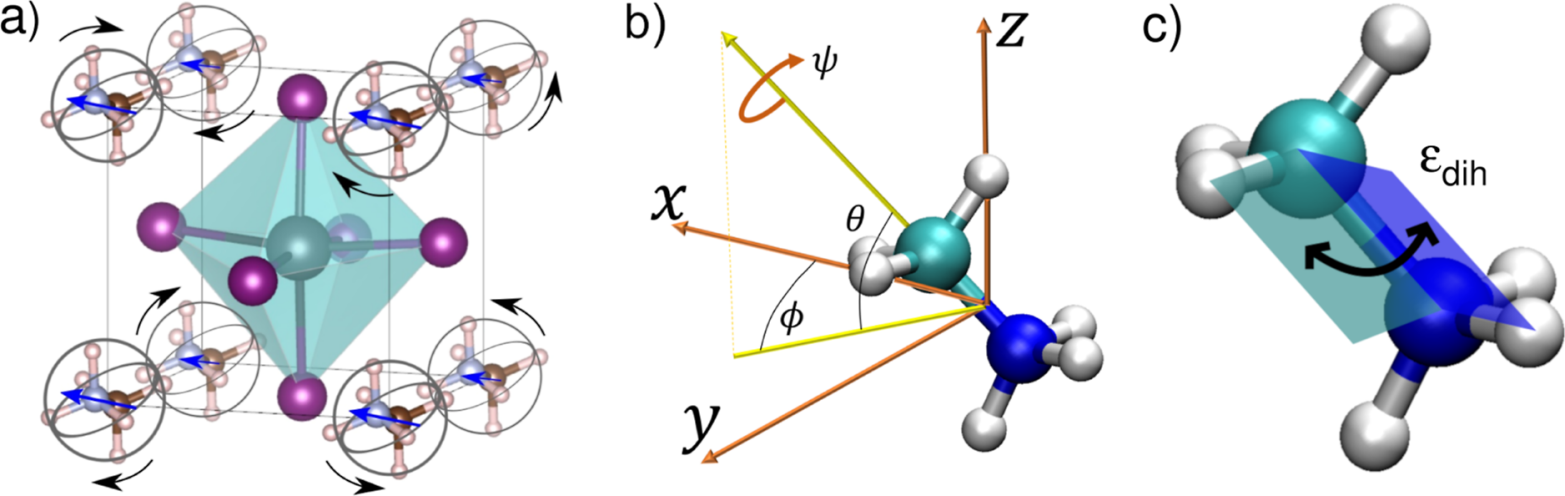
MAPI unit cell and MA molecule angular coordinates. (a) Sketch of MAPI in the high-temperature cubic perovskite phase. MA molecules are orientationally disordered. (b) Angles definition for describing the orientation of MA molecules. Angles θ and ϕ describe the orientation of the molecular C–N axis. Angle ψ refers to the rotation of the molecule around its C–N axis. (c) Dihedral angle describing the conformations of a MA molecule. Hydrogen, carbon, and nitrogen atoms are represented with white, green, and blue spheres, respectively.

Classical MD simulations in the *NpT* ensemble (i.e., considering fixed number of particles, pressure, and temperature) have been conducted for bulk MAPI (Figure 1a) using the atomistic force field developed by Mattoni and collaborators.^37,38^ This classical interatomic potential is based on a hybrid formulation of the Lennard–Jones and Buckingham pairwise interaction models and describes the MA molecules through harmonic-bonded interactions. Long-range electrostatic interactions are also appropriately accounted for by this atomistic force field. The high accuracy of this classical interatomic potential in providing correct MA^+^ orientational configurations, as benchmarked by quantum first-principles simulations, has been previously demonstrated^30,39^ (although it should be noted that it appreciably underestimates the temperature of the phase transition analyzed in this study, which involves the tetragonal *I*4/*mcm* and cubic *Pm*3̅*m* phases of MAPI^30,35,36^). The technical details of our MD–*NpT* simulations can be found in the Methods section.

### 2.2 Reference Systems and Angles Definition

The orientation of each individual MA molecule has been determined in a fixed lab reference system through Euler angles θ_lab_, ϕ_lab_, and ψ_lab_ (Figure 1b). The angles θ_lab_ and ϕ_lab_ determine the orientation of each C–N molecular bond in the fixed reference frame. The angle ψ_lab_ describes the rotation of each molecule around its C–N molecular axis. In order to analyze possible molecular orientational correlations (see next sections), the relative orientations between MA molecules need to be tracked. For this end, a comobile reference system is set on an arbitrary molecule, and the orientation of the other molecules are referred to it through the angles θ_rel_, ϕ_rel_ and ψ_rel_. The origin of this comobile reference system is set at half the distance between the C–N atoms, with the *z* axis, pointing to the carbon atom, the *y* axis being perpendicular to the plane defined by the atoms N–C–H, and the *x* axis being perpendicular to the *y* and *z* axes. In addition, a dihedral angle, ε_dih_, formed by the intersecting planes containing the H–C–N and C–N–H* atoms (where H belongs to the molecular methyl group and H* to the ammonia group, Figure 1c), is used to monitor possible MA^+^ conformational changes.

### 2.3 Entropy Calculations

The entropy of the low-*T* ordered and high-*T* disordered phases of MAPI were determined as a function of temperature, *S*_total_, using the relation
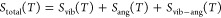
1where *S*_vib_ is the entropy contribution resulting from the atomic vibrations, *S*_ang_ from the molecular angular degrees of freedom (i.e., orientational and conformational), and *S*_vib-ang_ accounts for possible couplings between the vibrational and molecular angular degrees of freedom.

Likewise, the molecular angular entropy was assumed to be correctly evaluated with the expression
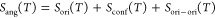
2where *S*_ori_ is the entropy contribution resulting from the orientational degrees of freedom of a single and noninteracting MA molecule, *S*_conf_ from the conformational changes of a single and noninteracting MA molecule, and *S*_ori-ori_ accounts for possible orientational correlations between different molecules.

In this study, we primarily have focused on a comparative analysis of the entropy of the low-*T* ordered and high-*T* disordered phases of MAPI at the corresponding *T*-induced phase transition point (other possible external fields such as hydrostatic pressure and electric bias are set to zero). Thus, the physical quantity that is quantitatively analyzed in detail here corresponds to the phase-transition entropy change, defined as
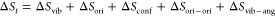
3where Δ*S*_*x*_ ≡ *S*_*x*_^disord^ – *S*_*x*_^ord^ and all the entropy terms are evaluated at the phase-transition temperature *T*_*t*_. The main improvement of this Δ*S*_*t*_ definition, compared to that in a previous work,^30^ is the inclusion of molecular conformational changes (Δ*S*_conf_), molecular orientational correlations (Δ*S*_ori-ori_), and possible couplings between the vibrational and molecular orientational degrees of freedom (Δ*S*_vib-ang_).

#### 2.3.1 Vibrational Entropy

The vibrational density of states (VDOS) ρ(ω), where ω represents a lattice vibration frequency, provides information on the phonon spectrum of a crystal and allows the estimation of key thermodynamic quantities like the vibrational free energy, *F*_vib_, and vibrational entropy, . A possible manner of calculating ρ(ω) from the outputs of MD–*NpT* simulations consists in estimating the Fourier transform of the velocity autocorrelation function (VACF),^30,40,41^ defined as
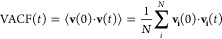
4where **v**_**i**_(*t*) represents the velocity of the *i*-th particle and ⟨···⟩ statistical average performed in the *NpT* ensemble. Subsequently, VDOS can be estimated as
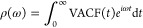
5which fulfills the normalization condition
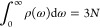
6with 3*N* being the number of real and positively defined phonon frequency branches of the crystal.

Upon determination of ρ, the vibrational free energy can be straightforwardly estimated with the formula^42^
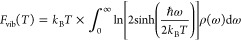
7where *k*_B_ is the Boltzmann’s constant. Consistently, the vibrational entropy adopts the expression
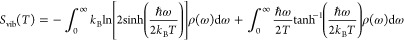
8

#### 2.3.2 Molecular Orientational Entropy

For a continuous random variable *x* with probability density *f*(*x*), its entropy is defined as:^43^
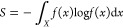
9where the integral runs over all possible values of *x*. If *x* represents a microstate characterizing a particular thermodynamic macrostate, then the following Gibbs entropy can be defined for the system of interest^44^
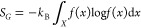
10

In an orientationally disordered crystal, molecules reorient in a quasi-random manner. By assuming the MA molecules in the HOIP crystal to be independent one from the other, one may estimate a probability density function for their orientation, *f*(θ_lab_, ϕ_lab_, ψ_lab_), from the atomistic trajectories generated during long MD–*NpT* simulations. In this case, the following three-dimensional molecular orientational entropy can be defined, under the implicit assumption that the length of the MA molecules remains fixed^45−47^
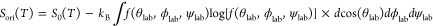
11where *S*_0_ is a reference entropy term. (For a fluid, the value of this reference entropy term matches that of an ideal gas system under the same temperature and density conditions as the system of interest;^45−47^ however, for an orientationally disordered solid, this reference term is not as straightforward to define).^30^

In practice, the calculation of *S*_ori_ entails the construction of histograms for which the continuous polar variables are discretized, {θ_lab_, ϕ_lab_, ψ_lab_} → {θ_lab,*i*_, ϕ_lab,*i*_, ψ_lab,*i*_}. Accordingly, one may define the bin probabilities^43^
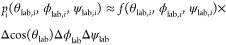
12where Δcos(θ_lab_) Δϕ_lab_ Δψ_lab_ is the volume of a histogram bin (selected to be constant in this study). Consistently, one can rewrite the molecular orientational entropy in the discretized form
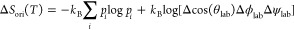
13where the value of the reference entropy term in eq 11 has been offset.

#### 2.3.3 Molecular Conformational Entropy

At finite temperatures, MA molecules may undergo conformational changes that contribute to the total entropy, *S*_conf_. The dihedral angle formed by the intersecting planes containing the H–C–N and C–N–H* atoms (where H belongs to the molecular methyl group and H* to the ammonia group, Figure 1c), ε_dih_, may be used to monitor such conformational molecular changes. Analogously to the case of the orientational entropy, a probability density function can be estimated for this molecular dihedral angle from the atomistic trajectories generated during long MD–*NpT* simulations, *f*(ε_dih_). Likewise, a bin probability can be defined, *p*_*i*_(ε_dih,*i*_) ≈ *f*(ε_dih,*i*_) Δε_dih_, leading to the conformational entropy expression
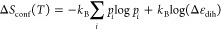
14where Δε_dih_ is the fixed length of a histogram bin and the value of the corresponding reference entropy term has been offset.

#### 2.3.4 Molecular Correlation Entropy

Equation 13 would provide the total orientational entropy for the MA molecules if these were completely independent of one from the other. However, neglecting molecular orientational correlations in HOIPs may lead to inaccuracies unless these correlations are rigorously quantified and demonstrated to be negligible. In this study, we account for the effects of molecular correlations in the entropy, *S*_ori-ori_, by considering up to second-order terms in the many-body expansion of the full orientational entropy.^45−47^ To this end, we monitor the relative orientation between pairs of molecules in a comobile reference system (Section 2.2) and calculate the molecular correlation entropy difference between the disordered and ordered phases like^45−47^
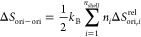
15where *n*_*i*_ is the number of molecules in the *i*-th spherical coordination shell (e.g., *n*_1_ = 6 and *n*_2_ = 12) and *n*_shell_ is the number of considered coordination shells (equal to 5 in the present study). The expression for the individual entropy terms *S*_ori,*i*_^rel^ is equivalent to that shown in eq 13, making the angular substitutions {θ_lab_, ϕ_lab_, ψ_lab_} → {θ_rel_, ϕ_rel_, and ψ_rel_}.

It is worth noting that a simulation approach for the calculation of entropy terms, similar to the one introduced in this work, has been employed in two previous studies on the molecular crystals LiCB_11_H_12_^48^ and C_5_H_12_O_2_.^49^ In both cases, we observed consistently good agreement for the total phase-transition entropy change when compared to results obtained using alternative thermodynamic approaches, such as the Clausius–Clapeyron (CC) equation and direct calculation of the internal energy difference from MD–*NpT* simulations (see Section 3.6 for further details of these methods). Thus, despite the limitations in performing additional benchmark calculations to further validate the accuracy of our entropy estimates for MAPI (e.g., harmonic and thermodynamic integration approaches^50−52^), due to its highly anharmonic and orientationally disordered nature, we are confident in the reliability and numerical precision of our simulation study.

## 3 Results and Discussion

In a previous work,^30^ we already estimated the phase-transition entropy change for MAPI associated with the temperature-induced order–disorder phase transition occurring near room temperature (i.e., between the low-*T* tetragonal *I*4/*mcm* and high-*T* cubic *Pm*3̅*m* phases^35,36^) through MD–*NpT* simulations by considering vibrational and molecular orientational degrees of freedom. Nevertheless, in this study, we present several critical improvements to our original Δ*S*_*t*_ calculation, which can be generalized to other HOIPs and solids exhibiting molecular orientational disorder (e.g., plastic crystals^48,53−56^). These critical computational improvements include considering entropy contributions from molecular conformational changes (Δ*S*_conf_), molecular orientational correlations (Δ*S*_ori-ori_), and possible couplings between the vibrational and molecular orientational degrees of freedom (Δ*S*_vib-ang_) (Section 2.3). Next, we present and discuss the calculation of Δ*S*_*t*_ term-by-term, comment on the potential formation of ordered MA^+^ nanodomains, and make insightful connections with technological applications. Our main entropy numerical findings are summarized in Table 1.

**Table 1:** Contributions to the Total Entropy Change Associated with the *T*-Induced Order–Disorder Phase Transition in MAPI, Δ*S*_*t*_^a^

	Δ*S*_vib_	Δ*S*_ori_	Δ*S*_conf_	Δ*S*_ori-ori_	Δ*S*_vib-ang_	Δ*S*_*t*_
(J K^–1^ kg^–1^)	+21.7	+13.5	≈0	+3.8	–12.6	+26.4
(%)	+82	+51	≈0	+15	–48	+100

a Δ*S*_*x*_ ≡ *S*_*x*_^disord^-*S*_*x*_^ord^ and all the entropy terms are evaluated at the phase-transition temperature, *T*_*t*_. Δ*S*_vib_ represents contributions from the lattice vibrations, Δ*S*_ori_ from the MA orientational degrees of freedom, Δ*S*_conf_ from the MA conformations, Δ*S*_ori-ori_ from the molecule–molecule orientational correlations, and Δ*S*_vib-ang_ from the couplings between the vibrational and molecular angular degrees of freedom.

### 3.1 Vibrational Entropy

The simulation results presented in this section have been mostly reported in a previous work of ours.^30^ These previous numerical findings are reproduced here for coherence and completeness reasons.

**Figure 2:**
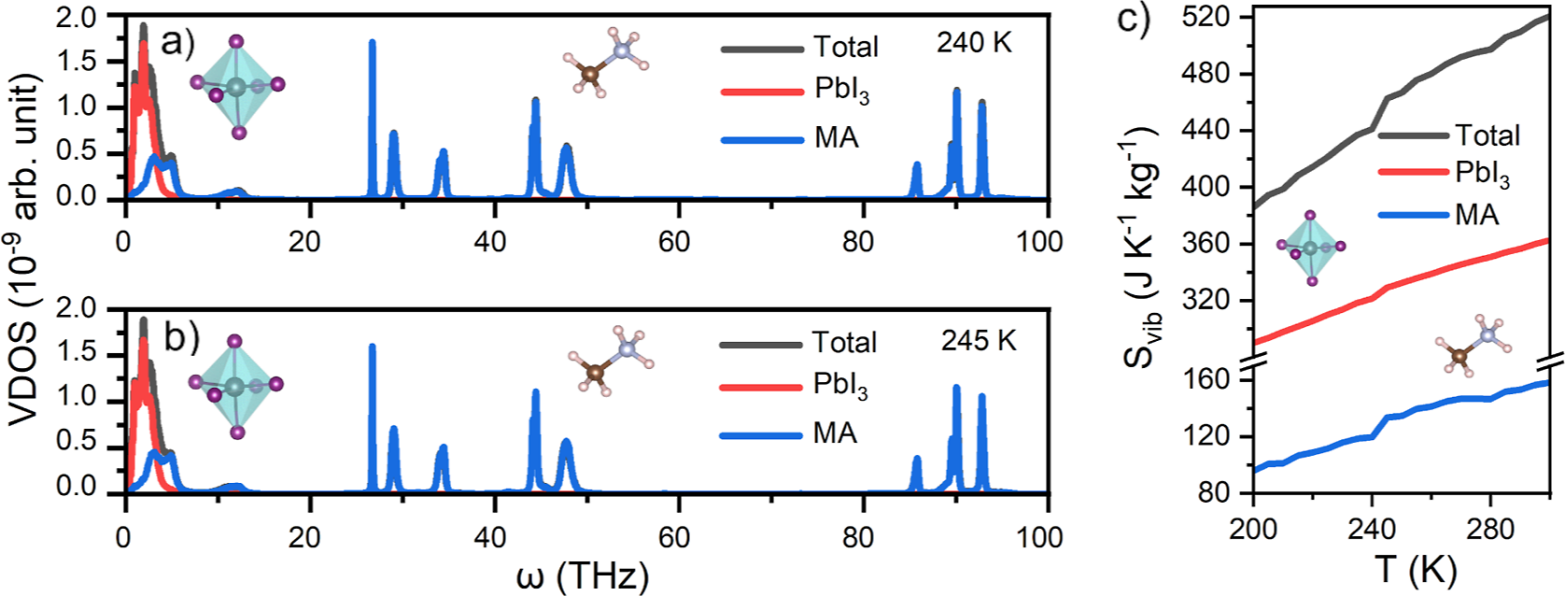
VDOS and vibrational entropy (*S*_vib_) of MAPI calculated at different temperatures. VDOS results were obtained (a) for the ordered phase at *T* = 240 K and (b) for the disordered phase at *T* = 245 K. (c) Vibrational entropy of MAPI expressed as a function of temperature.

Figure 2 shows the VDOS and partial organic and inorganic contributions estimated for MAPI at temperatures slightly above and below the simulated order–disorder phase transition points (i.e., 245 and 240 K). The VDOS contribution corresponding to the inorganic part, namely, the PbI_3_ octahedra, is clearly dominant in the low-frequency range of 0 ≤ ν ≤ 5 THz (Figure 2a–b). This result follows from the fact that the lighter atoms, which typically vibrate at higher frequencies, entirely reside in the organic molecules. Consistently, the range of moderate and high frequencies, ν ≥ 5 THz, is mostly governed by MA cation vibrations.

**Figure 3:**
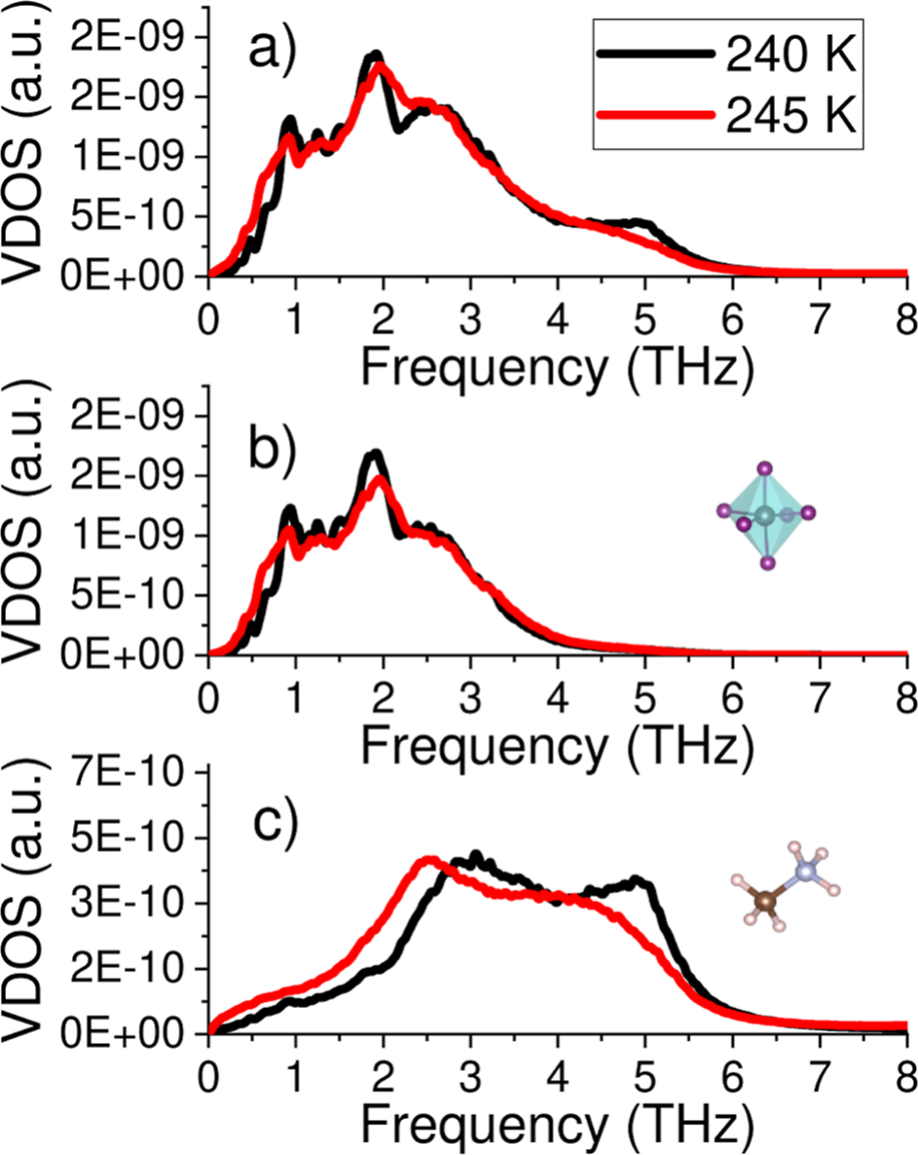
VDOS of MAPI in the low-frequency range. (a) Total VDOS for the ordered phase at *T* = 240 K and disordered phase at *T* = 245 K. (b) Inorganic contribution to the total VDOS for the ordered phase at *T* = 240 K and disordered phase at *T* = 245 K. (c) Organic contribution to the total VDOS for the ordered phase at *T* = 240 K and disordered phase at *T* = 245 K. To improve the visualization, a change of scale has been applied on the coordinate axis of this figure.

Albeit the VDOS enclosed in Figure 2a–b may seem quite similar at first glance, there are significant differences among them (Figure 3a). In particular, the high-*T* disordered phase accumulates more phonon modes in the low-frequency range 0 ≤ ν ≤ 2 THz than does the low-*T* ordered phase. This effect has a strong influence on the vibrational entropy of the system, *S*_vib_, which is significantly larger for the high-*T* disordered phase. It is worth noting that in previous first-principles computational studies,^57,58^ the low-frequency vibrations of the PbI_3_ octahedra (Figure 3b) were associated with bending and stretching modes, while the low-frequency phonon modes of the MA cations were linked to a combination of libration and translation (Figure 3c).

Figure 2c shows the *S*_vib_ values estimated as a function of temperature. A clear surge in vibrational entropy is observed at the phase-transition point, Δ*S*_vib_ = +21.7 J K^–1^ kg^–1^. The positive sign indicates that the vibrational entropy of the high-*T* disordered phase is larger than that of the low-*T* ordered phase. The primary contribution to this vibrational entropy increase comes from the molecular cations, which is equal to +13.9 J K^–1^ kg^–1^ and almost twice as large as that calculated for the inorganic part (namely, +7.8 J K^–1^ kg^–1^). Thus, although the low-frequency range in VDOS is dominated by the inorganic anions, the organic MA cations have a larger influence on the vibrational entropy change associated with the order–disorder phase transition, Δ*S*_vib_.

### 3.2 Molecular Orientational Entropy

To estimate the orientational entropy associated with the order–disorder phase transition of MAPI, Δ*S*_ori_, we have extended the computational approach introduced in a previous work of ours^30^ to include the three Euler angles that fully determine the orientation of an arbitrary (and rigid) MA cation, namely, {θ, ϕ, ψ} (Figure 1b). To calculate Δ*S*_ori_, we initially assumed the MA cations to be independent of each other. Consequently, the three Euler molecular angles should be referred to the stationary lab reference system, namely, {θ_lab_, ϕ_lab_, and ψ_lab_} (Section 2.2). The complete molecular orientation maps thus correspond to three-dimensional probability density functions (pdf) considering those three angles.

**Figure 4:**
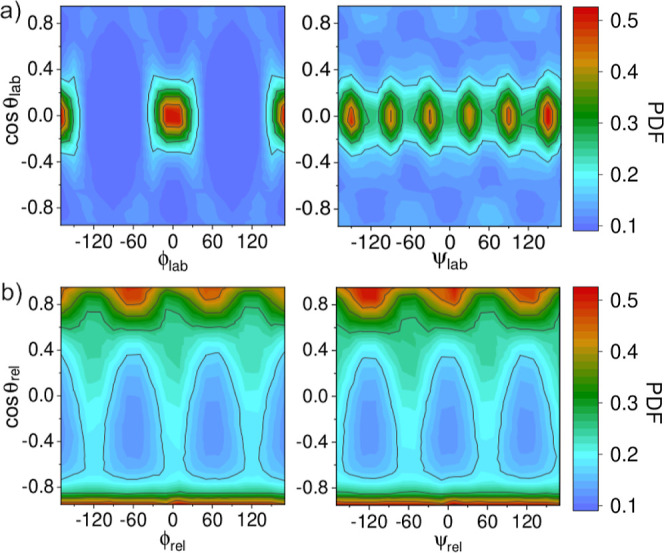
Bivariate angular probability density function (pdf) for MA molecules in the low-*T* ordered phase. Results were obtained at *T* = 240 K in the (a) lab-fixed (“lab”) and (b) molecule-mobile (“rel”) reference systems. For the “rel” case, the six MA cations within the first coordination shell were considered. Red, green, and blue colors represent high-probability, medium-probability, and low-probability configurations, respectively.

**Figure 5:**
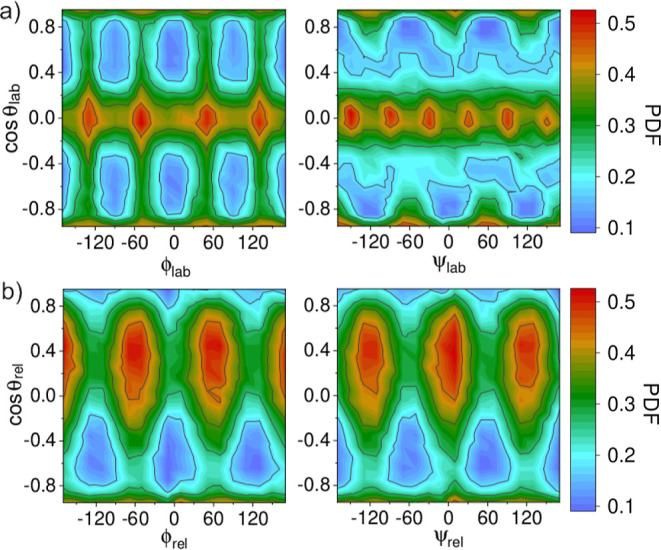
Bivariate angular probability density function (pdf) for MA molecules in the high-*T* disordered phase. Results were obtained at *T* = 260 K in the (a) lab-fixed (“lab”) and (b) molecule-mobile (“rel”) reference systems. For the “rel” case, the six MA cations within the first coordination shell were considered. Red, green, and blue colors represent high-probability, medium-probability, and low-probability configurations, respectively.

In the previous work,^30^ the ψ_lab_ angle, which accounts for the rotation of the MA molecules around their C–N molecular axis (Figure 1b), was not considered, thus potential entropy contributions resulting from this angular degree of freedom were totally neglected. Results for the full MA orientational pdf calculated for the low-*T* ordered and high-*T* disordered phases of MAPI, as projected onto two different planes, are shown in Figures 4a and 5a, respectively.

As shown in Figure 4a, in the low-*T* ordered phase, the MA cations do not reorient since the equilibrium molecular orientations, represented by bright spots in the cos(θ_lab_)–ϕ_lab_ pdf, are disconnected (i.e., angular transition paths connecting them are absent). This outcome was anticipated and is consistent with our previous results.^30^ On the other hand, as expected, in the high-*T* disordered phase, the molecular C–N axis are free to reorient, as indicated by the regions of nonzero probability appearing between the equilibrium molecular orientations (Figure 5a). For this phase, and also in agreement with our previous findings,^30^ there a six possible equilibrium molecular orientations: two “apical” [cos(θ_lab_) = ± 1, ϕ_lab_ = 0] and four “equatorial” [cos(θ_lab_) = 0, ϕ_lab_ = ± 60, 120°].

Figures 4a and 5a also show the pdf associated with the azimuthal angle ψ_lab_, which describes the rigid rotation of the MA cations around their C–N axis (Figure 1b). Interestingly and surprisingly, it is found that for both the low-*T* ordered and high-*T* disordered phases of MAPI, there are rotational paths connecting high probability regions (i.e., equilibrium molecular orientations). This result implies that close to the transition temperature the molecules rotate around their C–N axis in both the ordered and disordered states. It is worth noting that this type of azimuthal orientational disorder does not break the symmetry of the crystal (i.e., the corresponding equilibrium configurations are equivalent among them) and hence cannot be resolved in diffraction experiments.^59−62^ This class of molecular disorder, present in both the low-*T* ordered and high-*T* disordered phases of MAPI, is consistent with previous results reported in experimental neutron scattering works.^22,63^ Nevertheless, in our simulation study, in contrast to experiments, we are able to clearly distinguish between rotations of the MA molecule as a whole around the C–N axis and conformational molecular changes involving independent rotations of either the ammonia or methyl group (see next section).

The orientational phase-transition entropy change obtained from the two-dimensional pdf associated with the two angles {cos(θ_lab_), ϕ_lab_} amounts to +10.7 J K^–1^ kg^–1^.^30^ However, when considering the full orientational pdf associated with the three Euler angles {cos(θ_lab_), ϕ_lab_, and ψ_lab_}, it is found that Δ*S*_ori_ = +13.5 J K^–1^ kg^–1^. Therefore, by including the molecular azimuthal degree of freedom, the orientational entropy change increases by approximately 25%, thus representing a substantial correction. It is noted in passing that the degree of disorder associated with the molecular azimuthal angle ψ_lab_ is larger for the high-*T* phase.

It is instructive to compare the Δ*S*_ori_ value obtained from the atomistic MD–*NpT* simulations with that estimated straightforwardly from symmetry and simplified thermodynamic arguments. From the cos(θ_lab_)–ϕ_lab_ pdf shown in Figures 4a and 5a, it is observed that the MA cations can adopt two possible orientations in the low-*T* ordered phase and six in the high-*T* disordered phase. This elementary counting leads to a rough orientational entropy change of *k*_B_(ln6-ln2), which equals 14.7 J K^–1^ kg^–1^ and is approximately 40% larger than the correct Δ*S*_ori_ value obtained directly from the atomistic simulations. We may thus conclude that such a rough estimation of the orientational entropy change, although it appears to be a reasonable initial guess, is quantitatively not accurate.

### 3.3 Molecular Conformational Entropy

**Figure 6:**
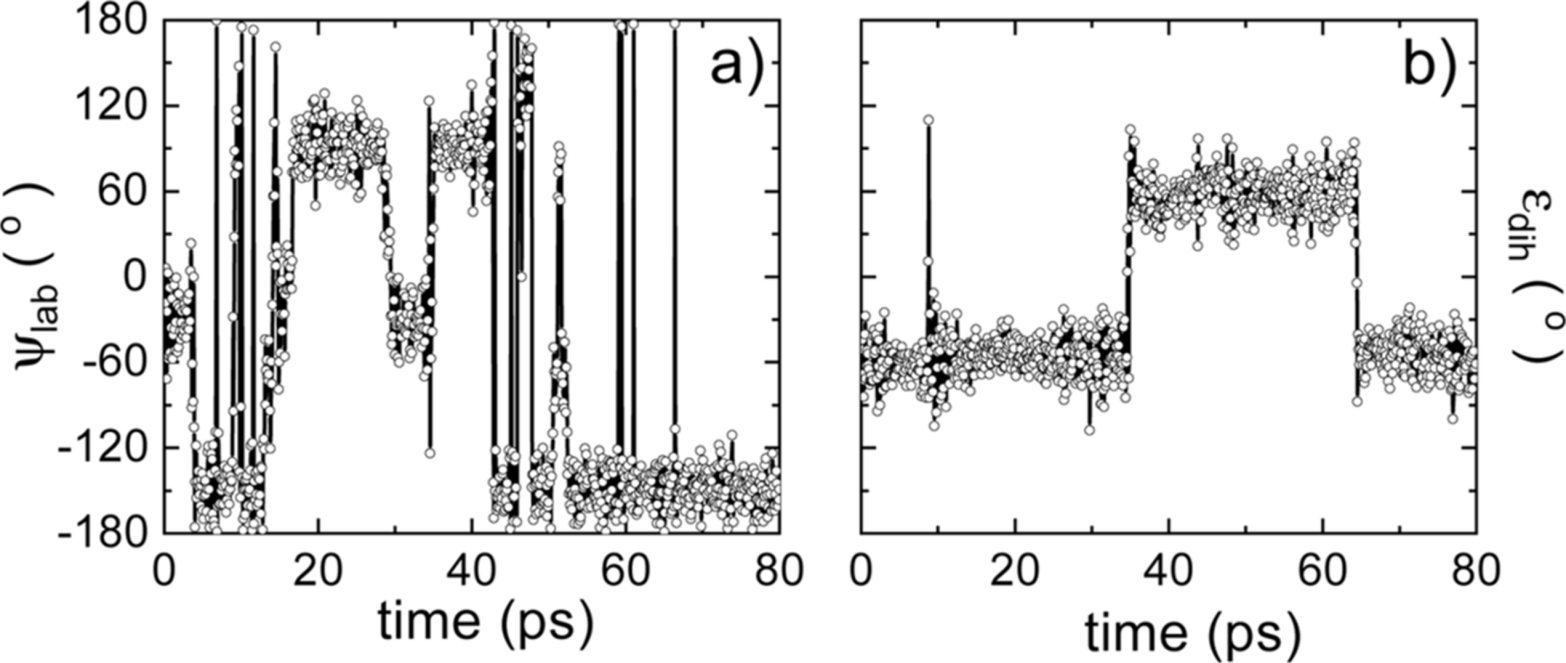
Dynamical description of the MA cation orientation around its C–N axis and molecular conformations for the low-temperature ordered phase. Time evolution of (a) the azimuthal angle ψ_lab_ describing the MA^+^ rotation around its C–N axis and (b) the dihedral angle ε_dih_ describing molecular conformation (Figure 1).

**Figure 7:**
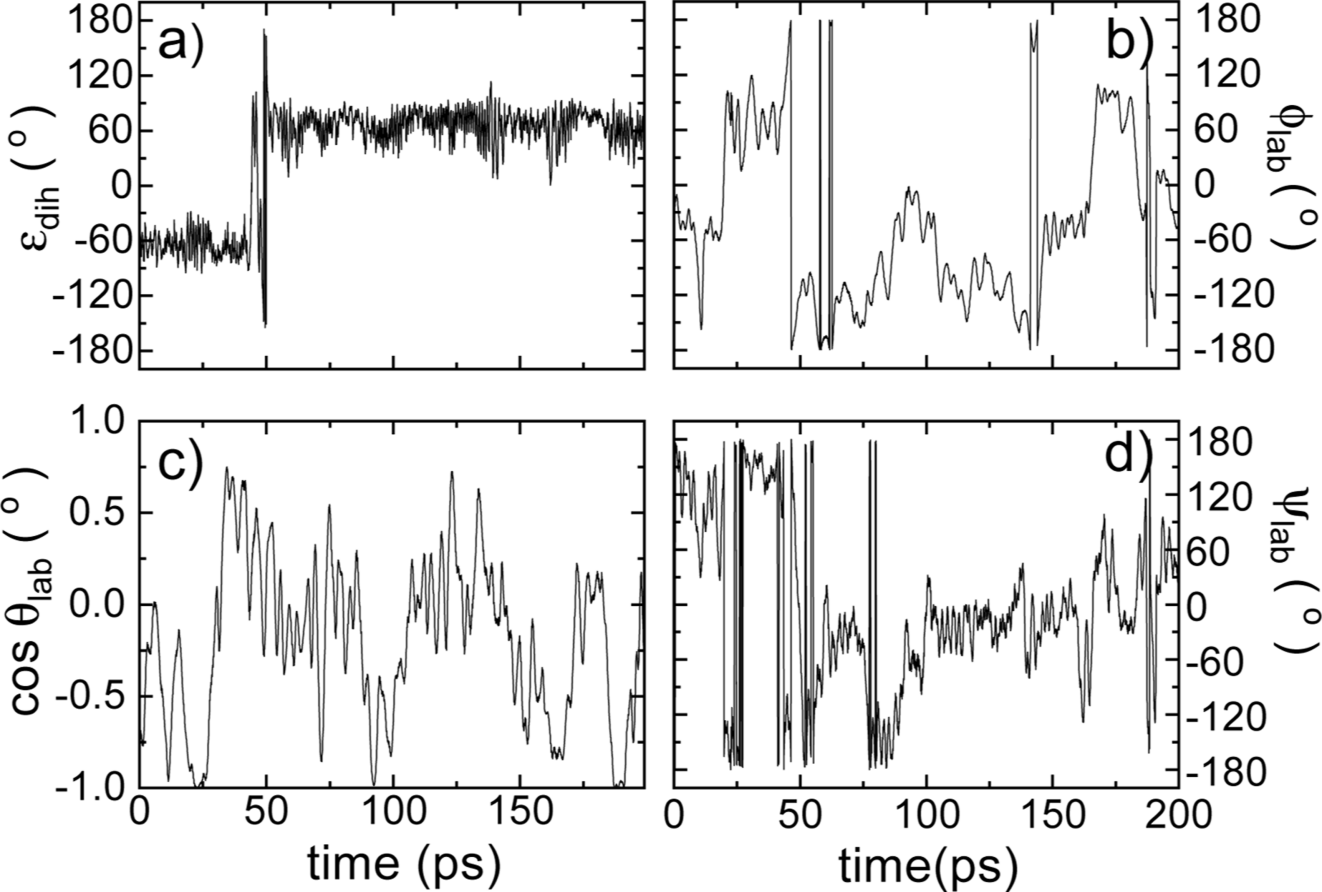
Dynamical description of the MA cation orientation around its C–N axis and molecular conformations for the high-temperature disordered phase. Time evolution of (a) the dihedral angle ε_dih_ describing molecular conformation, and the rest of MA angles (b) ϕ_lab_, (c) θ_lab_, and (d) ψ_lab_ describing molecular orientation (Figure 1).

In the previous section, we asserted that in both the low-*T* ordered and high-*T* disordered phases of MAPI, the MA cations perform rigid rotations around their molecular C–N axis, which do not affect the symmetry of the crystalline lattice. However, changes in the azimuthal angle ψ_lab_ could, in principle, also be due to conformational changes of the MA cation associated with disconnected rotations of the methyl (CH_3_) and/or ammonia (NH_3_) groups at the ends of the molecule. To check this possibility, we plot in Figure 6 and 7 the time evolution of the molecular angles {ψ_lab_, ε_dih_} for the low-*T* ordered and high-*T* disordered phases, respectively, where ε_dih_ is the dihedral angle formed by the intersecting planes containing the H–C–N and C–N–H* atoms (H belonging to the molecular methyl group and H* to the ammonia group, Figure 1c). For the sake of completeness, we also represent the dynamical evolution of the angles θ_lab_ and ϕ_lab_ for the high-*T* disordered phase in Figure 7.

For the low-*T* ordered phase (Figure 6), it is clearly observed that the sequence of ψ_lab_ and ε_dih_ changes occurring over time are uncorrelated. For instance, the dihedral angle changes twice its value within the time interval of 80 ps, from −60 to +60° and vice versa (Figure 6b), while the azimuthal angle changes several tens of times its value, −180° ≤ ψ_lab_ ≤ + 180°, within the same time interval (Figure 6a). A very similar behavior is also appreciated for the high-*T* disordered phase (Figure 7a,d). Consequently, it can be safely concluded that owing to the absence of time correlations between the angles ψ_lab_ and ε_dih_ in both phases, changes in the azimuthal angle correspond to rigid molecular rotations around the C–N axis. We note in passing that the dynamics of the dihedral angle is extremely slow in both the low-*T* ordered and high-*T* disordered phases, which denotes the high rigidity of the MA^+^ molecules.

**Figure 8:**
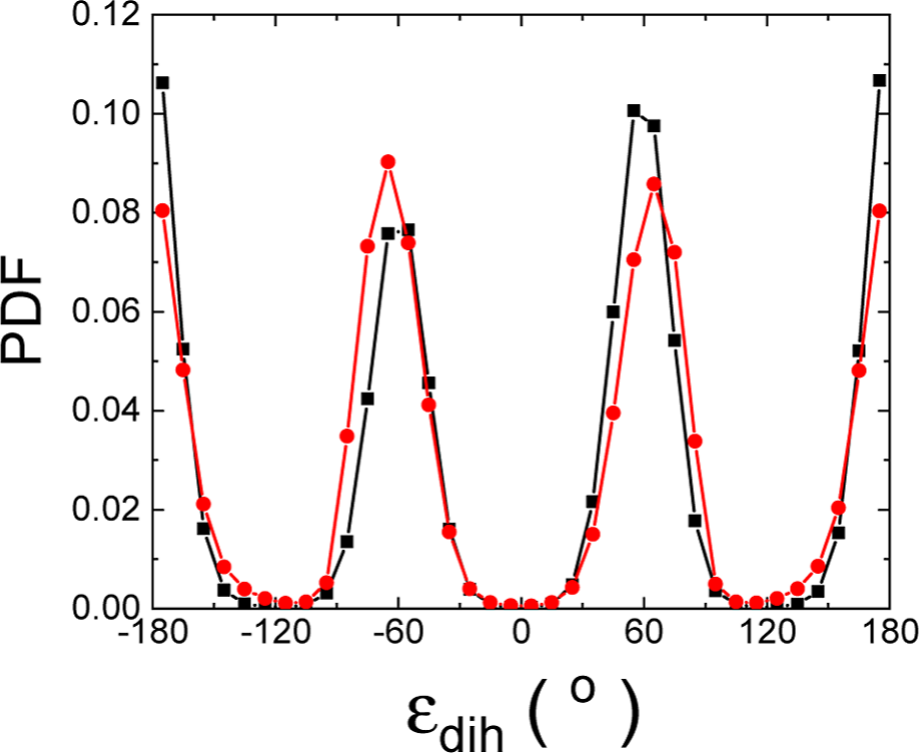
Angular probability density function (pdf) for the molecular dihedral angle ε_dih_ describing the conformation of MA molecules. Results are represented for the low-*T* ordered (red circles) and high-*T* disordered (black squares) phases. Solid lines are guides to the eye.

Figure 8 shows the pdf estimated for the molecular dihedral angle ε_dih_ for both the low-*T* ordered and high-*T* disordered phases of MAPI, close to the phase-transition temperature. It is observed that ε_dih_ essentially adopts equilibrium values ±60 and ±180° in both phases. Additionally, the estimated pdf are very similar for the two phases. As a consequence, the phase-transition entropy variation associated with molecular conformational changes (eq 14) turns out to be practically null for MAPI, namely, Δ*S*_conf_ ≈ 0. It can be concluded then that the MA cation presents high rigidity in both the low-*T* ordered and high-*T* disordered phases and that below and close to room temperature molecular conformational disorder in MAPI is strongly inhibited.

### 3.4 Molecular Correlation Entropy

Figures 4b and 5b show the pdf estimated for the three Euler angles {θ_rel_, ϕ_rel_, ψ_rel_} describing the relative orientation between closest MA cations (i.e., within the first coordination shell) in a comobile molecular reference system (Section 2.2) for the low-*T* ordered and high-*T* disordered phases of MAPI, respectively. Using these orientational probability maps and others involving successive coordination shells, it is possible to quantify molecular ordering and the phase-transition entropy change associated with the molecule–molecule correlations, as explained in Section 2.3.4.

In the low-*T* ordered phase (Figure 4b), the relative orientation between the closest molecules is most likely for cos(θ_rel_) = ± 1, which translates into parallel and antiparallel C–N bond arrangements or, equivalently, parallel and antiparallel molecular dipoles. Interestingly, it is observed that these maximum probability regions differ in shape. The reason for this asymmetry is the following. Two out of the six closest MA cations are parallel [cos(θ_rel_) = +1], located in the “tail” and “head” positions of the central MA^+^, and four are antiparallel [cos(θ_rel_) = −1], positioned in the equatorial plane of the central MA^+^. The molecules parallel to the reference MA cation slightly tilt their C–N axis relative to the central one, to change their ψ_rel_ orientation, causing cos(θ_rel_) to slightly depart from +1. This behavior, however, is not observed for the antiparallel molecules.

In the high-*T* disordered phase (Figure 5b), strong orientational correlations between the closest MA molecules are evident, as otherwise much more uniform and symmetric probability maps would have been obtained. For example, molecular parallel arrangements are strongly suppressed at high temperatures, as shown by the very low probability estimated for cos(θ_rel_) = +1. In this case, the most likely relative MA^+^ orientations correspond to three different molecular arrangements resulting in cos(θ_rel_) ≈ 0.4, which compensates for the total dipole moment deriving from the also highly probable antiparallel configuration [cos(θ_rel_) = −1]. It is worth noting that the number of equilibrium relative orientations is the same for the low-*T* ordered and high-*T* disordered phases, totaling four. The most significant molecular orientation difference between the two phases is a shift in the maximum probability from θ_rel_ ≈ 0° at low temperatures to θ_rel_ ≈ 66° at high temperatures.

In view of the fact that the reorientational MA^+^ motion in MAPI is correlated, we calculated the entropy difference between the low-*T* ordered and high-*T* disordered phases resulting from the molecular correlations at the corresponding phase-transition temperature, Δ*S*_ori-ori_ (Section 2.3.4). A positive (negative) Δ*S*_ori-ori_ value would indicate larger (smaller) molecular correlation entropy in the high-*T* disordered phase and consequently less (more) correlated molecular orientational dynamics in that phase. Adding consecutive entropy terms up to the fifth coordination shell (i.e., a maximum radial distance of 14.7 Å and 56 molecules), we estimated Δ*S*_ori-ori_ = +3.8 J K^–1^ kg^–1^. It was found that this molecular correlation entropy was already numerically converged to within 0.1 J K^–1^ kg^–1^ at the fourth coordination shell, which involves a maximum radial distance of 13.2 Å and 32 molecules. The calculated Δ*S*_ori-ori_ value is positive, suggesting more substantial correlations in the molecular orientational dynamics of the low-*T* phase. Moreover, Δ*S*_ori-ori_ is comparable in magnitude, for instance, to the phase-transition entropy gain associated with the azimuthal angle ψ_lab_ (Section 3.2) and hence is not negligible.

### 3.5 Molecular Dipoles Ordering

**Figure 9:**
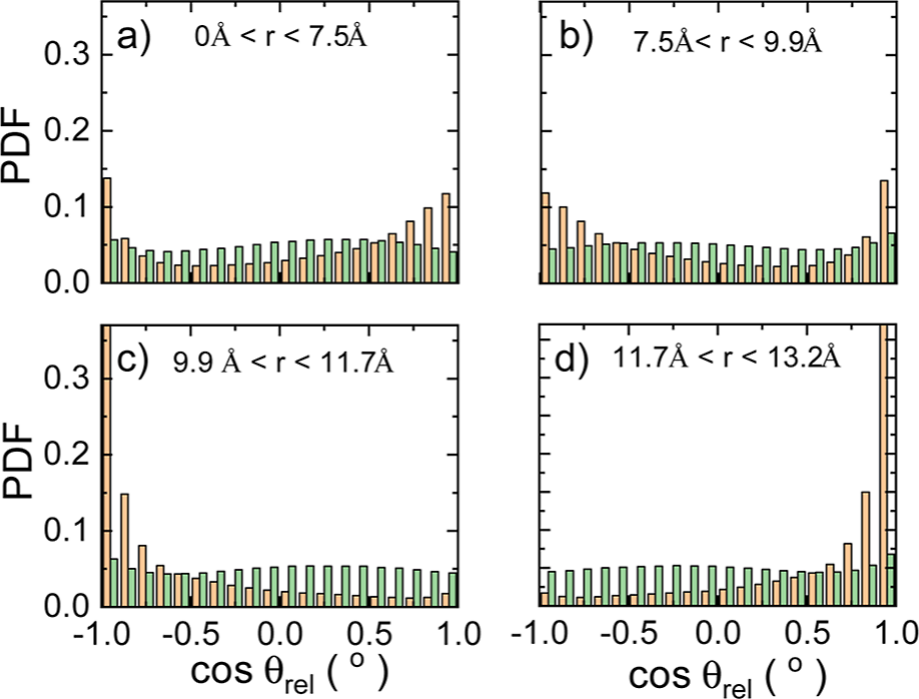
Probability density function (pdf) for the molecular dipole orientation in the coMobile (“rel”) reference system obtained across successive coordination shells. Results obtained for (a) first, (b) second, (c) third, and (d) fourth coordination shells. Orange and green bars represent results obtained for the low-temperature ordered and high-temperature disordered phases, respectively.

To further elucidate the molecular orientational disorder in MAPI and evaluate the possible formation of ordered MA^+^ nanodomains at finite temperatures, we represent in Figure 9 the pdf estimated for the angular quantity cos(θ_rel_) across successive coordination shells (i.e., from the first up to the fourth). Although our primary focus is on the molecular structure of the high-*T* disordered phase, we have also included pdf results for the low-*T* ordered phase in Figure 9 to facilitate the interpretation and comparison of our findings.

It is clear from Figure 9 that the relative orientation between molecules in the high-*T* disordered phase follows a fairly uniform probability distribution within all of the analyzed coordination shells, implying that there is no particularly preferred MA^+^ orientation. For instance, the average value of the angular pdf within the first coordination shell (Figure 9a) amounts to ⟨ cos(θ_rel_)⟩ = 0.02, with similar values obtained for the other consecutive coordination shells. Conversely, the pdf of the low-*T* ordered phase exhibits well-defined peaks within all of the coordination shells at cos(θ_rel_) = ± 1, as expected (Section 3.4), leading to general antipolar ordering.

**Figure 10:**
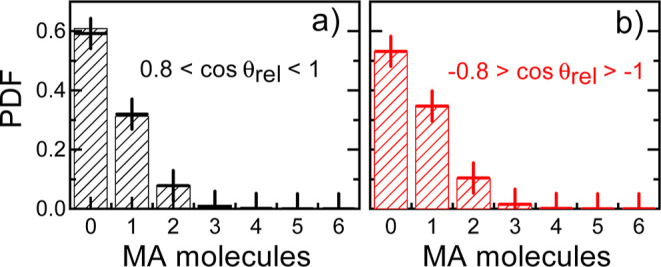
Probability distribution function (pdf) for the number of molecules in the first coordination shell of a MA cation that are parallel and antiparallel to it. Results were obtained by averaging over all molecules and simulation time. Pdf corresponding to (a) parallel and (b) antiparallel relative molecular dipole orientations (black and red boxes). The crosses in the figures represent analogous results obtained for a random distribution of relative molecular dipole orientations.

Interestingly, a very subtle alternation from marginally parallel to marginally antiparallel relative molecular arrangements is observed for the high-*T* disordered phase as the radial distance increases. However, this slight variation of relative MA^+^ orientations developing through successive coordination shells does not induce the appearance of nanoregions with well-defined polarization. This outcome is illustrated in Figure 10, where we compare the pdf estimated for the number of parallel and antiparallel MA cations to a chosen one within the first coordination shell, as directly obtained from our MD–*NpT* simulations, and consider completely random, and thus uncorrelated, relative molecular orientations. No appreciable differences are observed between the two pdfs. It can then be concluded that the appearance of polar nanodomains in MAPI is precluded since the MA molecules tend to orient homogeneously throughout the crystal. A similar behavior has been recently reported for a plastic crystal.^64^

### 3.6 Vibrational–orientational Correlation Entropy

There is an additional source of potential entropy variation in MAPI related to the correlations between the molecular angular and vibrational degrees of freedom, Δ*S*_vib-ang_ [eq 3], which mainly results from the interdependencies between the organic and inorganic ions. Unlike the rest of the terms in eq 3, there is neither an analytical expression nor a computational recipe to directly evaluate Δ*S*_vib-ang_. Nevertheless, this particular entropy variation can be indirectly estimated at the order–disorder phase-transition point using simple thermodynamic arguments and MD–*NpT* simulations, as we explain below.

At the order–disorder phase-transition temperature, the Gibbs free energy (*G* ≡ *H* – *TS*, where *H* stands for enthalpy) of the low-*T* ordered and high-*T* disordered phases of MAPI should be equal. Therefore, it follows that Δ*S*_*t*_ = Δ*H*_*t*_/*T*_*t*_. Since our MD–*NpT* simulations are performed at zero pressure, the enthalpy difference between the high-*T* disordered and low-*T* ordered phases are equal to their internal energy difference at the phase-transition temperature, namely, Δ*H*_*t*_ = Δ*U*_*t*_. This latter quantity can be directly obtained with great precision from the MD–*NpT* simulations and, consequently, Δ*S*_*t*_ as well. Knowing the value of all the entropy terms in eq 3 except Δ*S*_vib-ang_, it follows that
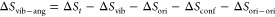
16

From our MD–*NpT* atomistic simulations, we obtained Δ*S*_*t*_ = +26.4 J K^–1^ kg^–1^ for the *T*-induced order–disorder phase transition occurring in MAPI. This value is in fairly good agreement with an equivalent estimation reported in work,^30^ Δ*S*_*t*_^CC^ = +28.4 J K^–1^ kg^–1^, which was based on the Clausius–Clapeyron (CC) method and is therefore more prone to numerical errors. Using eq 16 and the phase-transition entropy change values reported in previous sections, we conclude that Δ*S*_vib-ang_ = −12.6 J K^–1^ kg^–1^ (Table 1). The size of this vibrational–orientational correlation entropy is roughly 50% of the total phase-transition entropy change, which is surprisingly large and comparable in absolute value to the entropy change contribution Δ*S*_ori_ resulting from the individual MA^+^ orientational degrees of freedom (Section 3.2). This source of entropy change in MAPI, and arguably in HOIP in general, has not been considered in previous studies but, given its large magnitude, should not be neglected.

Interestingly, the sign of the estimated Δ*S*_vib-ang_ is negative, unlike the rest of the entropy terms in eq 3. Consequently, the crossed vibrational–orientational contribution decreases the total phase-transition entropy change. From a physical point of view, the negative sign of Δ*S*_vib-ang_ can be understood in terms of the couplings between the vibrational and molecular angular degrees of freedom, which are substantially reinforced in the high-*T* disordered phase (i.e., a smaller entropy is identified here with a higher degree of concertation). This reasoning is consistent with the large increase in vibrational entropy observed across the order–disorder phase transition (Section 3.1). In particular, correlations extending over a large number of atoms, both organic and inorganic, may lead to low-frequency phonon modes due to the large effective mass associated with such lattice vibrations (recall that for harmonic oscillations, ω ∝ *m*^–1/2^).^48^ Additionally, the overlap between the PbI_3_ and CH_3_NH_3_ lattice excitations also increases in the disordered phase, particularly in the limit of very low frequencies (Figure 3), thus suggesting a larger degree of organic–inorganic concertation in the high-*T* phase.

### 3.7 Connections to Technological Applications

Table 1 reports the value of the different contributions to the total entropy change associated with the *T*-induced order–disorder phase transition occurring in MAPI, as well as their relative percentage. The three entropy variations Δ*S*_vib_, Δ*S*_ori_, and Δ*S*_vib-ang_ are found to be the largest in absolute value and similar in size, with the peculiarity that only the sign of the crossed vibrational–orientational entropy change is negative. In what follows, we make connections between our atomistic simulation findings and a few technological applications in which HOIPs are used or have been proposed as promising, namely, solid-state cooling, photovoltaics, and energy storage.

Solid-state cooling is an energy efficient and ecologically friendly technique with potential for solving the environmental problems posed by conventional refrigeration technologies relying on compression cycles of greenhouse gases.^65−67^ Upon moderate magnetic, electric, or mechanical field variations, auspicious caloric materials experience large adiabatic temperature variations (|Δ*T*| ∼ 1–10 K) due to phase transformations entailing large isothermal entropy changes (|Δ*S*| ∼ 10–100 J K^–1^ kg^–1^). HOIP have shown great promise as caloric materials because their order–disorder phase transition can be driven near room temperature under the influence of various external bias like hydrostatic pressure and electric fields.^11−14,29,30,34,68^

However, the entropy changes associated with the order–disorder phase transition in HOIP (|Δ*S*| ∼ 10 J K^–1^ kg^–1^) are about an order of magnitude smaller than those measured in plastic crystals (|Δ*S*| ∼ 100 J K^–1^ kg^–1^), a related family of materials composed exclusively of organic molecules. Plastic crystals like neopentilglycol (C_5_H_12_O_2_) have recently revolutionized the field of solid-state refrigeration due to the huge latent heat associated with their molecular order–disorder phase transition (similar to that of MAPI), easy synthesis, low toxicity, and reduced economic cost.^48,53−56^ The physical reason for the difference in phase-transition entropy change between HOIP and plastic crystals may partly originate from the Δ*S*_vib-ang_ contributions in the former materials, which, as revealed in this study, tend to reduce Δ*S*_*t*_. Consequently, a potential rational design strategy to improve the caloric figures of merit of HOIP could be to minimize the crossed vibrational–orientational couplings through chemical and/or structural engineering.^69^

In solar cells, the dielectric function of the light-absorbing materials, ε, is of the upmost importance as it directly impacts the binding energy of excitons, *E*_bind_ (i.e., photogenerated electron–hole pairs that remain electrostatically bound). In particular, *E*_bind_ should be minimized to facilitate the dissociation of excitons into free charge carriers and prevent electron–hole recombination.^70^ According to the Wannier-Mott model *E*_bind_ ∝ 1/ε^2^,^71^ meaning larger (smaller) values of ε correspond to smaller (larger) exciton binding energies. In HOIP, lattice vibrations are intrinsically coupled with cation orientational motion, both of which are considered to influence the material’s optoelectronic performance.^21−26,28^ Phonons can significantly modulate the band gap, charge transport, and exciton dynamics.^72,73^ Cation orientational dynamics may affect properties such as ferroelectricity, ion transport, and the dielectric behavior of HOIP.^74−76^

In the specific case of MAPI, ε is strongly influenced by the phonons and molecular cation rotations, which both contribute to the lattice component of the dielectric constant in the high-*T* disordered phase,^77−79^ increasing it from 5 to 33.^26,80,81^ As a result, the excitonic binding energy of MAPI decreases from approximately 16 meV in the low-*T* ordered phase to 6 meV in the high-*T* disordered phase.^26,81^

In this study, we have theoretically demonstrated that the couplings between phonons and cation orientational dynamics in MAPI are substantial and crucial for evaluating the entropy change associated with their *T*-induced order–disorder phase transition. Furthermore, by calculating the entropy difference term Δ*S*_vib-ang_, we have determined that vibrational–orientational couplings are stronger in the high-*T* disordered phase compared to those in the low-*T* ordered phase. This enhancement of the molecular cation–ionic anion interactions in the orientationally disordered phase may partially explain the observed increase in ε and the corresponding decrease in *E*_bind_. Consequently, a potential rational design strategy to reduce exciton binding energy in light-absorbing HOIPs, thereby mitigating detrimental electron–hole recombinations, could involve enhancing their vibrational–orientational couplings, either through chemical modifications and/or nanostructuring.^69^ A possible descriptor for quantifying the degree of interplay between organic cation orientation and octahedral anion vibration in the high-*T* disordered phases of HOIPs, could be the overlap between low-frequency lattice phonons with prevalent molecular and ionic character.^52^

Energy storage materials are crucial for powering vehicles, buildings, and portable devices in the push for clean energy. Lithium-ion batteries dominate the market, offering high energy density, low self-discharge, a negligible memory effect, high open-circuit voltage, and durability. Supercapacitors, another key class of energy storage devices, stand out with rapid charge/discharge rates, high power density, and long cycle lifetimes. Their capacity surpasses conventional capacitors, and their faster discharge enables quick electric vehicle charging. Interestingly, halide perovskite materials, originally designed for solar cells, have proven effective in energy storage due to their excellent ion diffusion properties (e.g., an experimentally measured room-temperature ionic conductivity of 10^–8^ S cm^–1^ for MAPI^82^). While ion diffusion was initially considered detrimental to solar cell performance, it is advantageous for lithium batteries and supercapacitors, enhancing their efficiency in energy storage applications.^8−10^

In MAPI, ionic diffusion, which is sustained by the presence of intrinsic defects (e.g., vacancies), is substantial and increases rapidly with rising temperature in the high-*T*, orientationally disordered phase.^38^ In this work, we simulated pristine MAPI systems (i.e., with no defects), thus purposely excluding ionic transport. Nevertheless, recent studies in the context of solid-state electrolytes have emphasized the significant role of the vibrating nondiffuse crystal matrix in ion migration.^40,41,83−86^ In particular, the interplay between mobile atoms and the anharmonic lattice dynamics of the host framework may enhance superionicity.

In this study, the high anharmonicity of the orientationally disordered phase of MAPI has been evidenced by a large accumulation of low-frequency phonon lattice modes (Figures 2–3) and a substantial vibrational contribution to the total phase transition entropy change, which amounts to approximately 80% (Table 1). Potential proxies for identifying good ionic conductors among HOIPs may thus include the average phonon frequency, which should be as low as possible, along with the lattice heat capacity and vibrational entropy, which should be as high as possible.^41^ Nonetheless, it remains to be determined how the cross-vibrational-orientational entropy change term, Δ*S*_vib-ang_, is affected by the presence of crystalline defects and superionicity in order to further elucidate the mechanisms of ionic transport in HOIPs. These, and other related, interesting questions will be analyzed in detail in future works.

## 4 Conclusions

A comprehensive analysis of the orientational disorder and molecular correlations in the archetypal hybrid organic–inorganic perovskite (HOIP) MAPI has been presented, relying on atomistic MD–*NpT* simulations and advanced entropy calculations. The main findings of our computational research are the following. Both in the low-*T* ordered and high-*T* disordered phases, there is dynamical orientational disorder associated with rigid MA^+^ rotations around the molecular C–N axis. This previously overlooked orientational degree of freedom positively contributes to the total phase-transition entropy change, Δ*S*_*t*_. The correlations between MA cations are substantial, especially in the low-*T* ordered phase, and have a sizable augmenting effect on Δ*S*_*t*_. Conformational molecular changes, on the other hand, are relatively infrequent in both low-*T* ordered and high-*T* disordered phases and do not appreciably contribute to the phase-transition entropy change. Interestingly, the couplings between the vibrational and orientational degrees of freedom are strengthened in the high-*T* disordered phase and have a substantial decreasing effect on Δ*S*_*t*_. Lastly, molecular correlations in the high-*T* disordered phase are predominant but markedly local; thus, the formation of nanoregions with a well-defined polarization is strongly suppressed. These fundamental outcomes may be tentatively generalized to other HOIP and have important ramifications for advanced energy and optoelectronic applications relying on this family of materials.

## 5 Methods

### 5.1 MD Simulations

We used the LAMMPS simulation code^87^ to perform systematic classical MD simulations in the *NpT* ensemble for bulk MAPI using the force field developed by Mattoni and co-workers.^37,38^ The average temperature was set using a Nosé–Hoover thermostat with a mean fluctuation of 5 K. The simulation box contained 3072 atoms (equivalent to 256 MAPI unit cells), and periodic boundary conditions were applied along the three Cartesian directions. The long-range electrostatic interactions were calculated by using a particle–particle particle-mesh solver to compute Ewald sums up to an accuracy of 10^–4^ kcal mol^–1^ Å^–1^ in the atomic forces. The cutoff distance for the evaluation of the potential energy was set to 12 Å. To determine the phase-transition temperature of MAPI at zero pressure, we conducted comprehensive *NpT*-MD simulations in the temperature range 180 ≤ *T* ≤ 340 K, taken at intervals of 10 K. In our MD–*NpT* simulations, the temperature steadily increased up to a targeted value of over 1 ns. Subsequently, the system was equilibrated at that selected temperature for 4 ns. The production runs then lasted for about 1 ns with Δ*t* = 0.5 fs, from which the velocities of the atoms and other key quantities (e.g., the potential energy and volume of the system) were extracted. From the production MD–*NpT* runs, a total of 1000 equispaced configurations were retrieved to obtain uncorrelated structural data and generate accurate probability density functions (pdf).

### 5.2 Molecular Angular and Entropy Analysis

The MA^+^ angular degrees of freedom have been retrieved from the atomic configurations generated during the MD–*NpT* simulations with the help of the freely available and open-source software ANGULA.^88^ ANGULA is designed to automatically and unsupervisedly determine the angles defining the orientational structure of molecular disordered crystals from data files containing their atomic positions, both in the fixed “lab” and coMobile “rel” reference systems. Among its many capabilities, ANGULA can generate angular probability density maps and directional radial distribution functions directly from sequences of molecular configurations. In this study, the angular molecular entropy terms *S*_ori_, *S*_ori-ori_, and *S*_conf_ have been directly computed from the outputs of our MD–*NpT* simulations with ANGULA.^88^
